# P47 Delphi consensus on antimicrobial stewardship in wound care; an evidence-based paradigm shift towards infection prevention, early intervention and treatment using microbial-binding dressings

**DOI:** 10.1093/jacamr/dlag102.053

**Published:** 2026-06-26

**Authors:** Astrid Probst, Bodo Günther, Emma Woodmansey, Febe Bruwer, George Smith, Kevin Woo, Klarida Hoxha, Patricia Idensohn, Paulo Ramos, Vivek Lakshmanan, Windy Cole, Zhavandre van der Merwe

**Affiliations:** Kreiskliniken, Reutlingen GmbH, Reutlingen, Germany; Western University of Applied Sciences, Bergen, Norway; Surgical Department, Stord General Hospital, Helse Fonna Health Trust, Stord, Norway; Clinical & Scientific Solutions, York, UK; Cardiff University, Wales, UK; Protea Wound and Wellness, Johannesburg, South Africa; Hull York Medical School, York, UK; Queen’s University, Kingston, Canada; Curtin University, Perth, Australia; GVM Care & Research, Emilia Romagna, Italy; Comp Consulting Limited, Stratford-Upon-Avon, UK; ULS Póvoa de Varzim Vila do Conde, Portugal; Portuguese Wound Care Association, Gondomar, Portugal; European Wound Management Association; Department of Endocrinology and Diabetic Foot Surgery, Amrita Hospital, Cochin, India; Director of Wound Care Research, Kent State University College of Podiatric Medicine, Cleveland, OH, USA; Murdoch University, Perth, Western Australia. Advanced Wound Care Specialist, 4 Wounds Wound Care Practice, Pretoria, South Africa

## Abstract

**Background:**

Antimicrobial resistance (AMR) is a growing concern in surgical and hard-to-heal wounds, with reported inappropriate use of topical antiseptics, as well as systemic antibiotics. A recent survey found 41.8% of healthcare professionals used antimicrobial prophylactically, against recommendations, while 37.2% did not follow antimicrobial stewardship (AMS) guidance, indicating a potential gap in best-practice treatment. Wound infection can delay healing, increase complications and can rapidly progress to spreading or systemic infection, particularly in high-risk patients. Early diagnosis and appropriate treatment are essential for improved outcomes; however, intervention should be balanced with a reduction of inappropriate use of antiseptics and antibiotics to minimize AMR. Widely supported by clinical evidence, wound dressings with the hydrophobic coating, Dialkylcarbamoyl chloride (DACC), which bind bacteria and fungi, termed a microbial-binding dressing (MBD), offer a validated treatment option for infection prevention and control (IPC) in wounds. MBD exert microbial control by physically binding microbes which are subsequently removed during dressings changes, this mode of action does not rely on the release and bioavailability of active agents, therefore AMR development is not expected.

**Objectives:**

To provide evidence-based consensus statements on the role of MBDs to prevent, control and treat wound infection and to identify the potential for prophylactic and early intervention with MBDs to prevent or minimize progression of infection, supporting more appropriate use of antimicrobials in wounds.

**Methods:**

A pragmatic review of the literature was performed, with evidence level and certainty assessed (OCEBM and GRADE respectively). Evidence supporting the role of MBDs to reduce; microbial burden, surgical site infection (SSI), clinical signs of infection, antibiotic use, antiseptic dressing use, time to healing or complication rates was explored and summarized. Statements informed by this evidence base were developed. A modified Delphi method was used to gauge agreement and consensus with these statements according to the AGREE II criteria. Statements were scored using a Likert scale from 1 to 5, by a panel of 10 wound experts using up to three rounds of anonymous voting (two rounds remotely and one-round at an in-person meeting) as appropriate. Acceptance was considered a mean score ≥4.00 ± standard deviation ≤1.00.

**Results:**

The literature review returned 12 studies on surgical incisions and 17 on hard-to-heal wounds, varying in evidence level and certainty which informed the statements. Strong agreement was reached for 14 statements; nine in round one, two in round two and three in round three (in-person meeting), with one statement split into two prior to agreement. The statements covered three themes: challenges of wound infection and AMR; benefits of MBDs for infection prevention and control (IPC); and early IPC in future AMS strategies.

**Conclusions:**

The identified evidence and supporting statements advocate early intervention and first-line treatment using MBDs to remove microorganisms from a wound without exposure to active antimicrobials agents. Incorporation of MBDs into surgical and hard to heal wound protocols supports effective, early IPC and treatment, minimizing the potential for AMR and consequently strengthening AMS across wound care.

